# De Novo Terpenes Emitted from Juvenile Leaves of *Eucalyptus globulus* Labill. subsp. *globulus*

**DOI:** 10.3390/molecules30102234

**Published:** 2025-05-21

**Authors:** Anthony J. Winters, Charles H. Hocart, Jörg-Peter Schnitzler, Ina Zimmer, Mark A. Adams, Heinz Rennenberg, Jürgen Kreuzwieser, Claudia Keitel

**Affiliations:** 1 Plant Breeding Institute, The University of Sydney, Cobbitty, NSW 2570, Australia; 2 Research School of Biology, Australian National University, Canberra, ACT 2600, Australia; 3 College of Climate and Environment, Jinan University, Panyu District, Guangzhou 511443, China; 4 Innovation Base for Air Quality Science and Management for Guangdong, Hong Kong and Macao Great Bay Area, Guangzhou 511443, China; 5 Research Unit Environmental Simulation, Helmholtz Zentrum München, D-85764 Neuherberg, Germany; joergpeter.schnitzler@helmholtz-munich.de (J.-P.S.); ina.zimmer@helmholtz-munich.de (I.Z.); 6 Centre for Carbon Water and Food, School of Life and Environmental Sciences, The University of Sydney, Brownlow Hill, NSW 2570, Australia; mark.adams@sydney.edu.au (M.A.A.); claudia.keitel@sydney.edu.au (C.K.); 7 School of Science, Computing and Emerging Technologies, University of Swinbourne, Hawthorn, VIC 3122, Australia; 8 Institut für Forstbotanik und Baumphysiologie, University of Freiburg, D-79110 Freiburg, Germany; heinz.rennenberg@ctp.uni-freiburg.de (H.R.); juergen.kreuzwieser@ctp.uni-freiburg.de (J.K.); 9 Center of Molecular Ecophysiology (CMEP), College of Resources and Environment, Southwest University, No. 2, Beibei District, Chongqing 400715, China

**Keywords:** BVOC, terpene emissions, ^13^C incorporation, de novo synthesis, *Eucalyptus globulus*, PTR-MS, GC-MS

## Abstract

The contributions of de novo synthesis to terpene emissions from *Eucalyptus globulus* subsp. *globulus* were determined by fumigating branchlets with ^13^CO_2_ in a gas exchange system. Of more than thirty-four terpenes emitted by this species, only four, i.e., isoprene, iso-valeraldehyde, *cis*-ocimene, and trans-caryophyllene, incorporated ^13^C into the terpene carbon skeleton during the ~5–6 h experiment. ^13^C incorporation into isoprene and iso-valeraldehyde reached a maximum of ca. 82% of the carbon skeleton, similar to *cis*-ocimene, with a maximum of 77% ^13^C incorporation after ~2.5 h exposure to ^13^CO_2_. Only ca. 20% of carbon was labelled in trans-caryophyllene after 5–6 h. the incorporation of ^13^C was observed only in compounds emitted from leaves, and was not detected in either individual oil glands or in bulk leaf tissue. The results suggest the de novo synthesis of some terpenes (isoprene, *cis*-ocimene, trans-caryophyllene, and iso-valeraldehyde) and their emission is independent of emissions of terpenes stored in oil glands.

## 1. Introduction

Terrestrial vegetation is the dominant source of biogenic volatile organic compounds (BVOCs) in the atmosphere [1]. BVOCs play fundamental roles in atmospheric chemistry and physics, including in the formation of secondary organic aerosols, surface layer ozone, and atmospheric radicals [2,3,4]. Accurate estimates of emissions of these compounds are particularly important for constraining models of atmospheric chemistry and transport. Terpenes are the most important quantitative class of compounds emitted by plants, with isoprene (c. 500 Tg C y^−1^) and monoterpenes comprising the largest sources in this group (c. 160 Tg C y^−1^), followed by sesquiterpenes (about 30 Tg C y^−1^) [1,5].

Our understanding of the chemical and biochemical regulation of these emissions remains modest. Isoprene (C_5_H_8_) and monoterpenes (C_10_H_15_) are synthesised by enzymes of the methylerithritol phosphate (MEP) pathway in the chloroplast, whereas sesquiterpenes (C_15_H_24_) are formed in the mevalonate (MVA) pathway in the cytosol [6]. In addition to their importance for tropospheric chemistry, terpenes influence global warming via their effects on atmospheric methane (CH_4_). The unsaturated double bonds of terpenes are highly reactive, leading to significantly depleted hydroxyl concentrations in the lower troposphere. These, in turn, reduce rates of CH_4_ decomposition [7,8].

Short-term emissions of terpenes at the leaf-level are known to be regulated by environmental factors, such as light and temperature, that directly influence their production and storage in oil glands within leaves [9]. For terpenes, two sources of emission have been identified: direct emission as volatile products of recent biosynthesis (“de novo” biosynthesis), or emission from a pool of previously synthesised volatile oils that are stored in specific storage structures [10]. One or both terpene sources may operate at the leaf level. For example, the monoterpene α-pinene is emitted but not stored by *Quercus ilex* [11], while α-pinene emitted from *Picea abies* needles originates from both stored and recently synthesised sources [12].

Many eucalypt species develop specialised leaf oil glands [13], a trait common to many members of the *Myrtaceae* [14]. The scant evidence to date suggests that eucalypt oil glands are the major, possibly sole, sources of terpene emissions. Eucalypts were used as a model to develop a temperature-dependent emission algorithm for α-pinene and eucalyptol (1,8-cineole) [15,16]—a model that has been used extensively in leaf-level BVOC emission studies. However, there is no direct evidence that storage organs are the sole sources of terpene emissions. Large areas of the Australian continent are dominated by eucalypts and several eucalypt species are planted worldwide for fibre and timber production, particularly *E. globulus, E. camaldulensis* and *E. grandis* [17]. A more detailed understanding of the rates and regulation of BVOC emissions from eucalypts will help elucidate the responsible chemical and biochemical processes, as well as better inform physical models of atmospheric pollution [18].

Gas exchange studies that manipulate light and temperature generally provide the first indications of de novo synthesis, particularly in species that possess storage organs [19]. While straightforward in principle, such studies can provide ambiguous results, largely because physicochemical properties such as solubility or vapour pressure can mask the temperature-dependent nature of emissions. Niinemets et al. [20] pointed out that even plants without specialised storage structures still partition BVOC to different sites within the leaf tissue (which then act as a slow-release terpene pool). A fast-release terpene pool can be defined as one consisting of molecules that have not been partitioned in this way, and that are emitted directly into the atmosphere after synthesis. Emissions from storage structures depend mainly on temperature, although they are dependent on the saturated vapour pressure of individual terpenes or classes thereof [21]. For example, monoterpene emissions from coniferous trees are mainly calculated using temperature-dependent algorithms [15,22].

The fumigation of leaves with ^13^C-labelled CO_2_ provides unambiguous evidence for the incorporation of recently assimilated carbon into the emitted BVOC. This method has been widely applied over the last 20 years to a number of plant species [10,12,23,24,25]. Experiments on plants lacking specific storage organs have shown that 100% incorporation of the labelled carbon is never achieved [26,27,28,29,30] while other, non-labelled, carbon sources also feed in intermediates to terpene synthesis [23,25,31,32,33]. While efforts to partition emissions from stored and non-stored sources may thus be confounded [34], identification of the phenomenon remains an important first step in constraining emission estimates.

To determine whether the emitted BVOC in eucalypts originated from de novo synthesis and/or storage glands, leaves of *E. globulus* were fumigated with ^13^CO_2_ and the emitted BVOC were analysed for ^13^C incorporation. In addition, we identified the relationship between monoterpene synthase activity and the emission of monoterpenes that incorporated labelled carbon.

## 2. Results

### 2.1. Origin of ^13^C-Labelled Terpenes

**Figure 1:**
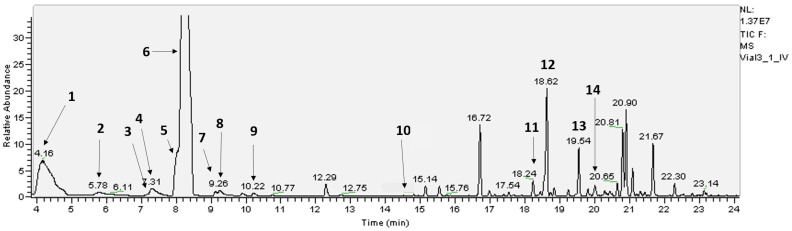
Typical GC-MS chromatogram of leaf oils of *E. globulus*. See Table 1 for the identity of the compounds, indicated in bold numbers, eluting under the respective peak.

Following six hours of ^13^CO_2_ fumigation under constant light (500 μmol m^−^^2^ s^−^^1^) and temperature (28 °C), only four terpenes detected by PTR-MS and GC-MS incorporated ^13^C. These terpenes were isoprene (C_5_H_8_), iso-valeraldehyde (C_5_H_10_O), *cis*-ocimene (C_10_H_16_, 1,8-cineole), and trans-caryophyllene (C_15_H_24_). All were collected from the gas phase. There was no evidence of ^13^C incorporation into any of the 34 terpenes isolated from either oil glands or whole-leaf samples (Figure 1, Table 1).

### 2.2. Identification and Kinetics of ^13^C-Labelled Compounds

^13^C-labelled compounds were identified with a combination of shifts in the isotopomer-signals of the PTR-MS and GC-MS analysis following desorption from charcoal cartridges. The monitoring of molecular and fragment ions of mono- or sesquiterpenes with PTR-MS could not be used to quantify the incorporation of ^13^C owing to their low abundance and the background emission of stored terpenes.

**Figure 2:**
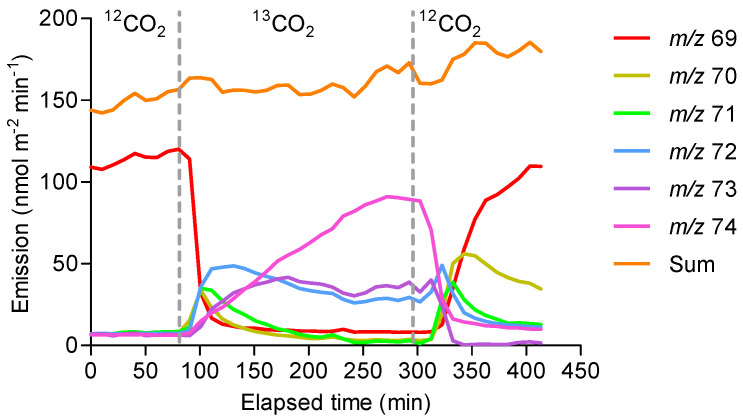
PTR-MS (SIM) monitoring of the time course of the ion intensities of ^13^C incorporation into isoprene emitted from the leaves of *E. globulus*. Ions were identified as follows: *m*/*z* 69 = ^12^C isoprene; *m*/*z* 70 = isoprene with one ^13^C atom; *m*/*z* 71 = isoprene with two ^13^C atoms, etc. The vertical dotted lines indicate switching between 1.1% and 99.5% ^13^CO_2_. Leaves were exposed at 28 °C under 500 μmol m^−^^2^ s^−^^1^ PAR for the duration of the experiment. The data represent a typical isotope profile representative of four replicates.

Isoprene was not detected by GC-MS (boiling point 34 °C) as the option of cryogenic focussing at the top of the column was not available. However, PTR-MS data showed ^13^C being incorporated into isoprene approximately 10 min after ^13^CO_2_ fumigation started (Figure 2), reaching a maximum incorporation of 82%, as shown by the intensity of *m*/*z* 74 ([MH]^+ 13^C_5_ isotopomer) after 2.5 h exposure to ^13^CO_2_. After reverting to CO_2_ at natural isotopic abundance, ^13^C incorporation declined, mirroring its incorporation after introduction.

**Figure 3:**
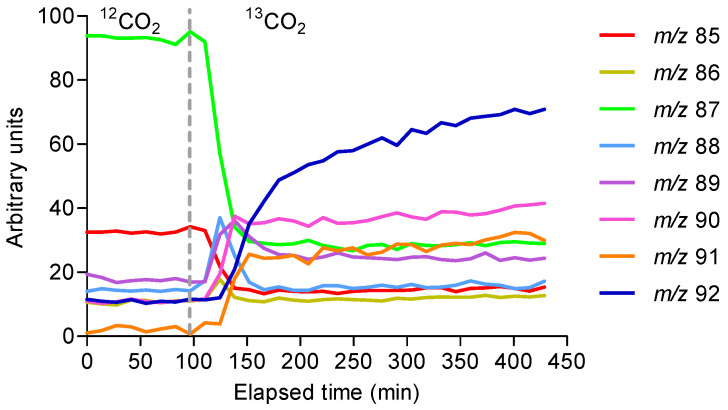
PTR-MS (SIM) monitoring the time course of the ion intensities of ^13^C incorporation for the *m*/*z* range 85–92. Ions at *m*/*z* 87 and *m*/*z* 85 declined in response to fumigation with ^13^CO_2_. Transient increases and decreases in the abundances of *m*/*z* 86, 88, and 89 are evident, as are sustained increases in *m*/*z* 91and *m*/*z* 92, indicating the incorporation of ^13^C. Evidence suggests that ^13^C is incorporated into a five-carbon BVOC, later identified as iso-valeraldehyde (C_5_H_10_O). Vertical dotted lines indicate switching between 1.1% and 99.5% ^13^CO_2_. Experimental conditions were identical to those in Figure 1. The data represent a typical isotope profile representative of four replicates.

**Figure 4:**
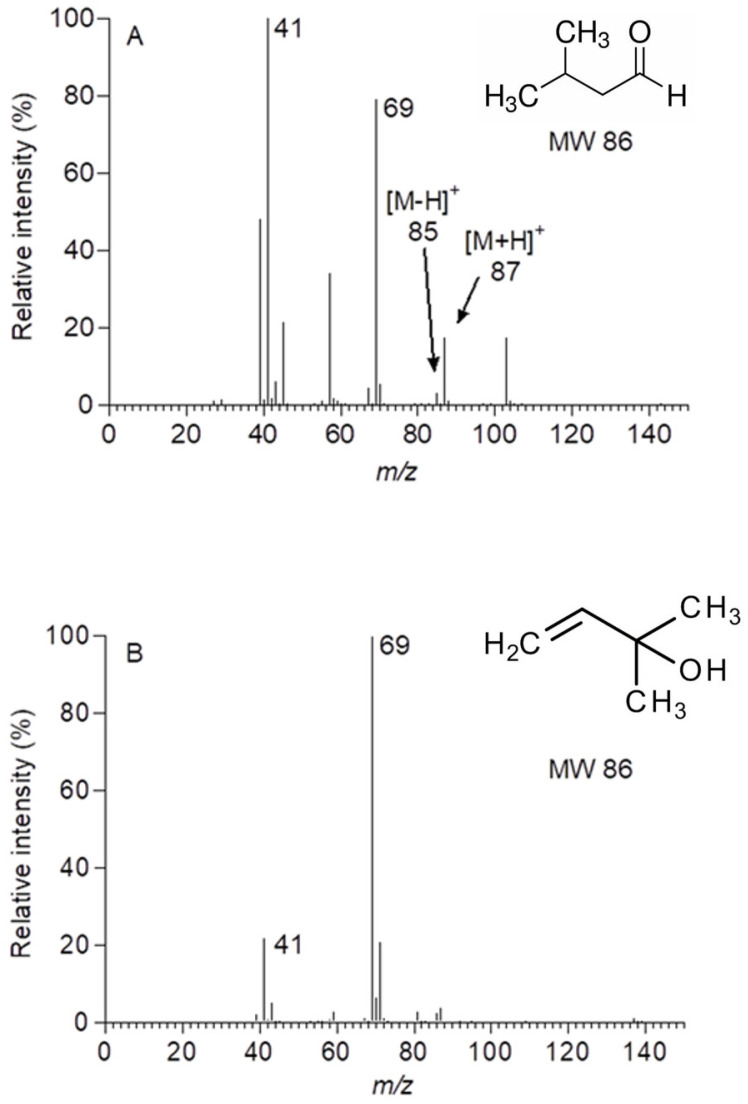
PTR-MS mass spectra of (**A**) iso-valeraldehyde and (**B**) 2-methyl-3-buten-2-ol (1 μL), diluted into individual 1 L Tedlar bags and filled with ultrapure N_2_. Spectra represent the background-subtracted average of 10–15 cycles of the PTR-MS in scan mode.

A second compound observed in the PTR-MS trace demonstrated similar ^13^C incorporation kinetics. ^13^C incorporation began about 15 min after fumigation started (Figure 3), reaching a maximum of 82% after 5 h (i.e., *m*/*z* 92, [MH]^+. 13^C_5_ isotopomer). It should be noted here that *m*/*z* 90 ions are a mixture of the ^13^C_5_ [M-H]^+^ and ^13^C_3_^12^C_2_ [MH]^+.^ isotopomers. The rates of ^13^C incorporation were slightly faster into isoprene (*m*/*z* 69, τ1/2 = 4.47 min) compared to the second compound (*m*/*z* 85, τ1/2 = 5.11 min; *m*/*z* 87, τ1/2 = 5.94 min). This finding was confirmed by monitoring the corresponding decreases in the intensity of both the [MH]^+^ ions (*m*/*z* 87) and the [M-H]^+.^ ions (*m*/*z* 85) representing the ^12^C_5_ isotopomer (Figure 4). Incrementally greater masses showed transient peaks following the sequential replacement of ^12^C with ^13^C atoms, akin to the labelling pattern of isoprene (Figure 2). Hence, the second molecule seems to be a C_5_ compound of molecular mass 86, with ionisation in the PTR-MS inducing the addition (*m*/*z* 87) and removal (*m*/*z* 85) of a single proton.

The molecular mass of 86 corresponds to iso-valeraldehyde and several methylbutenol isomers, all with the structural formulae C_5_H_10_O. Mass scans in the range *m*/*z* 81–95 ruled out monoterpenes (C_10_H_16_) as potential contributors, while isoprene could also be excluded given its lack of ion fragments at these masses. p-Cymene produces ion fragments at *m*/*z* 93 [35] (Winters, unpublished data) but showed no response to ^13^CO_2_ fumigation. Iso-valeraldehyde, detected previously in the analysis of leaf oils from a number of eucalypt species [36] and two alcohols—3-methyl-3-buten-1-ol, emitted by *Querus ilex* [27] and 2-methyl-3-buten-2-ol, emitted by a number of *Pinus* species [37,38,39]—were considered candidates given their common molecular mass and elemental composition. Qualitative gas standards, produced by the dilution of neat liquids into N_2_, were used to examine the fragmentation spectrums of each compound ionised by the PTR-MS. Iso-valeraldehyde produced fragments at *m*/*z* 85 and *m*/*z* 87 in similar proportions to those seen in the labelling study (Figure 4A), while 2-methyl-3-buten-2-ol did not (Figure 4B). Subsequent analysis by GC-MS after desorption from charcoal cartridges confirmed iso-valeraldehyde as the likely compound.

**Figure 5:**
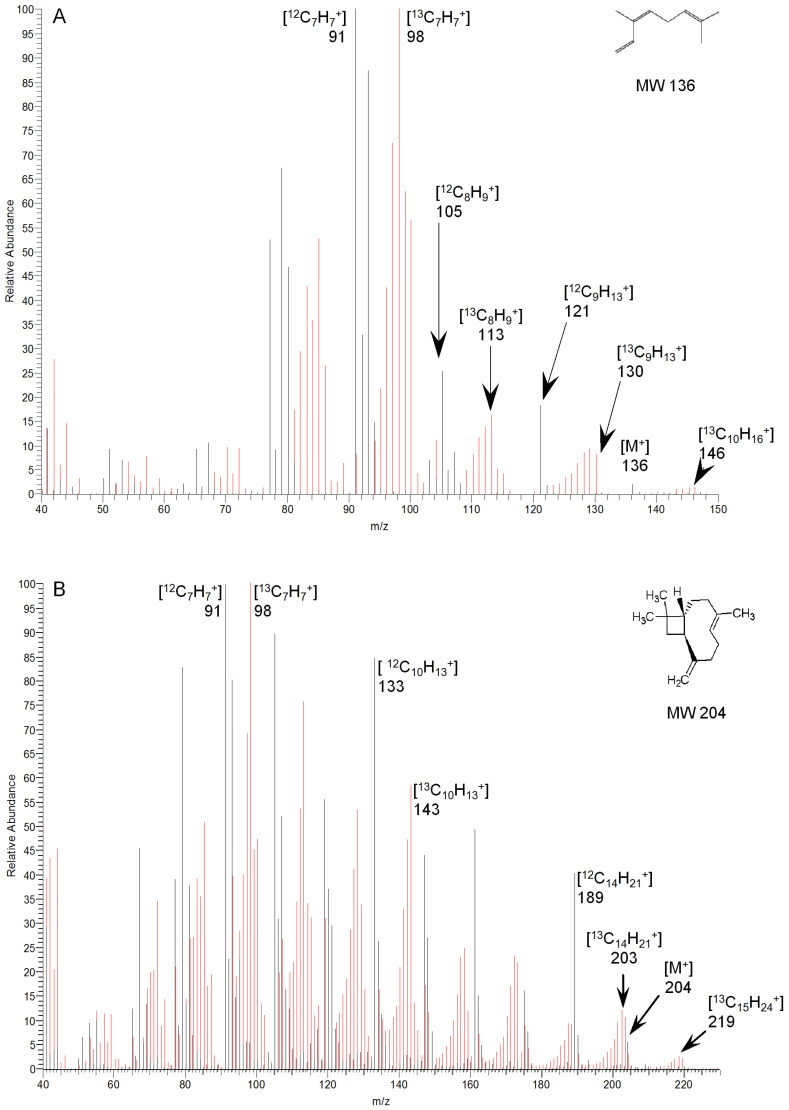
EI mass spectra from GC-MS analysis of (**A**) *cis*-ocimene and (**B**) trans-caryophyllene, emitted from leaves of *E. globulus,* showing a shift in fragmentation resulting from the incorporation of ^13^C. The ^13^C-labelled spectra (red) were captured 6 h after the onset of labelling and are compared with the unlabelled spectra (black) showing the natural isotopic abundance.

**Figure 6:**
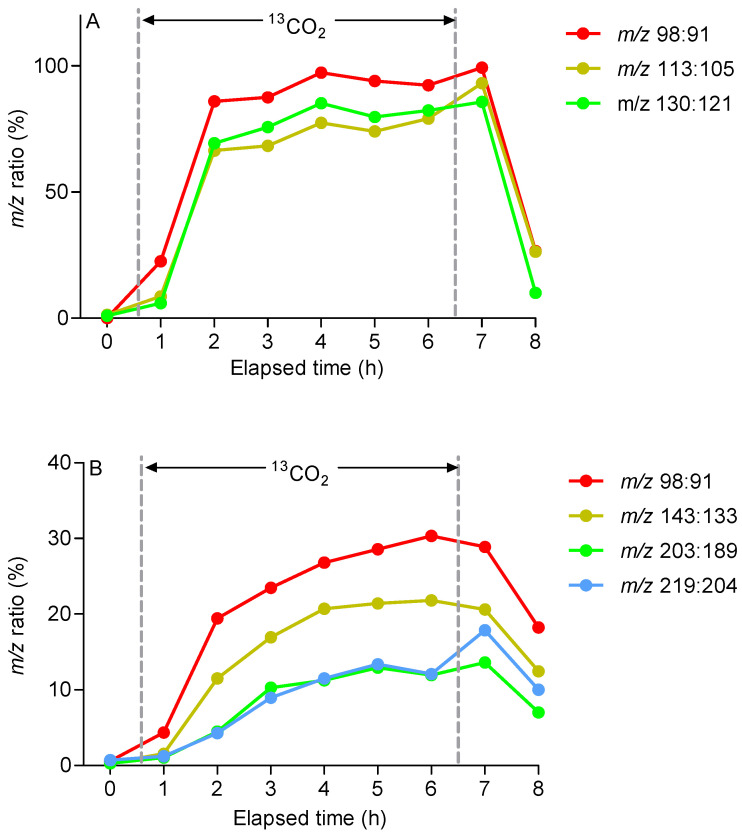
Time course of ^13^C incorporation into (**A**) *cis*-ocimene and (**B**) trans-caryophyllene emitted from leaves of *E. globulus*. The relative incorporation of ^13^C was calculated as [*m*/*z* (labelled)/(*m*/*z* unlabelled + *m*/*z* labelled)] × 100 using ions from EI mass spectra averaged across the total ion chromatogram for a given compound at each time point. The duration of ^13^CO_2_ fumigation (0.5 to 6.5 h) is indicated on each graph by the vertical dotted lines. Data are from a single tree but are typical of the pattern observed in four trees.

GC-MS (following desorption from charcoal cartridges) provided clear evidence for the incorporation of ^13^C into two further terpenes, the monoterpene (C10) *cis*-ocimene and the sesquiterpene (C15) trans-caryophyllene (Figure 5). An isomeric form of ocimene, tentatively identified as trans-ocimene through a comparison of the retention times and fragmentation patterns with an authentic standard, also incorporated ^13^C, but the compound was not consistently present in samples and was excluded from further analysis. The degree of the incorporation of labelled carbon into these C10 and C15 terpenes was determined through a comparison of the respective mass spectra over the course of the labelling experiment, with ions selected according to the abundance in the spectrum or their relationship to the molecular ion. Despite the low relative abundance (<2%) of the *cis*-ocimene molecular ion (M^+.^, *m*/*z* 136), a corresponding ion at *m*/*z* 146, representing the incorporation of ten ^13^C atoms, was clearly discernible (Figure 5A). This incorporation was confirmed through analysis of an [M-15]^+.^ ion (*m*/*z* 121; 18%) and the incorporation of nine ^13^C atoms could be clearly observed in the corresponding labelled ion at *m*/*z* 130. The incorporation of ^13^C into the [M-15]^+.^ ion reached a maximum of approximately 77% after 2.5 h (Figure 6A), similar to that reported for isoprene. The trans-caryophyllene molecular ion (M^+.^, *m*/*z* 204) had a 6% abundance and the incorporation of fifteen ^13^C atoms could be clearly observed in the corresponding ion at *m*/*z* 219 (Figure 6B). The incorporation of ^13^C into trans-caryophyllene appeared to stabilise at 15–20% after 5–6 h.

### 2.3. Monoterpene Synthase Activity

**Figure 7:**
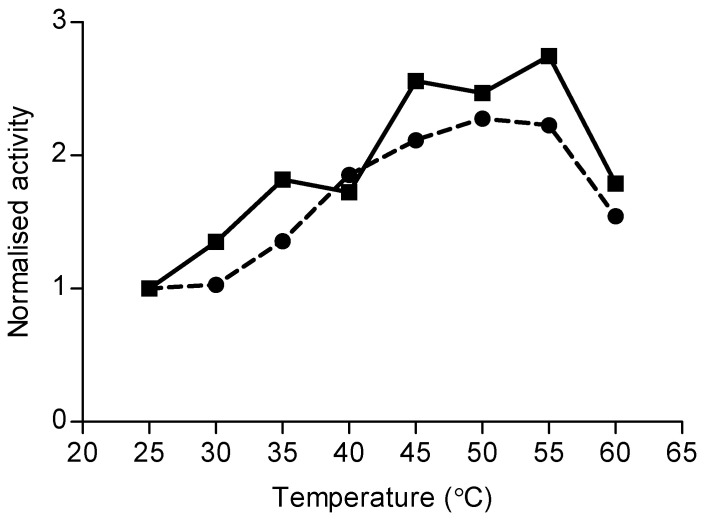
In vitro synthesis of *cis*-ocimene in response to incrementally higher incubation temperatures during the monoterpene synthase assay for leaves of *E. globulus* (squares) and *E. viminalis* (circles). Each symbol is the mean of two assays at the temperature indicated on the x-axis.

Mono-terpene synthase (TPS) activities were determined by headspace analysis following the incubation of protein extracts from eucalyptus leaves with geranyl diphosphate (GDP) [40]. Assays were problematic owing to stored oils being carried through the protein extraction process, as a headspace analysis of denatured (boiled) extracts produced similar results. Variations in the protocol, including salting out extracts and radioactive phosphatase assays using [1-^3^H]-GDP [40], failed to resolve the issue. However, incremental increases in incubation temperature resulted in increased production of *cis*-ocimene. This phenomenon was observed in leaf extracts of both *E. globulus* and *E. viminalis* (Figure 7). In both species, the maximum enzymatic production of *cis*-ocimene was recorded between 50 °C and 55 °C. Above this temperature range, activity declined (likely due to enzyme denaturation). Other monoterpenes, such as α-pinene, showed no response to temperature.

## 3. Discussion

The exposure of *E. globulus* leaves to ^13^CO_2_ suggests that isoprene and iso-valeraldehyde preferentially incorporated recently fixed carbon. In addition, the monoterpene *cis*-ocimene and the sesquiterpene trans-caryophyllene also incorporated recently synthesised carbon and appeared subject to regulation by light as well as temperature.

### 3.1. The Origin of Labelled Terpenes

The terpenes emitted by *E. globulus* likely originate from both oil glands and mesophyll tissue. The emissions of stored and de novo synthesised compounds are also compartmentalised at these two sites, as the absence of labelled carbon in compounds stored in oil glands argues against the possibility of synthesis in gland epithelial cells with the products deposited into the glands themselves. It seems unlikely that we lacked the detection power for labelled compounds in oil glands given the number of glands sampled and the sensitivity of our analysis. Additionally, labelled compounds were absent in whole-leaf tissue, i.e., they were absent from tissue outside oil glands. However, clearly labelled compounds were detected in leaf emissions, and given the similarity in labelling patterns among the three terpenes and iso-valeraldehyde, it seems likely that the chloroplasts of mesophyll cells are both a route for carbon fixation and terpene synthesis. Detailed biochemical analysis is needed to verify this circumstantial evidence. Nonetheless, an almost identical pattern to that reported here was found in Norway spruce and Scots pine following ^13^CO_2_ fumigation [10]. In contrast, ^14^C labelling in Maritime pine detected monoterpene synthesis in the epithelial cells of growing parts of needles, while older parts were significantly depleted in labelled carbon, and labelled sesquiterpenes were detected only in whole-leaf tissue surrounding resin ducts [41].

The ^13^C label appeared rapidly in isoprene following exposure to ^13^CO_2_, but the temporal resolution required the use of a charcoal adsorbent. Hence, the appearance of ^13^C in *cis*-ocimene and trans-caryophyllene could not be quantified as accurately as in isoprene. Nonetheless, both the time taken for ^13^C enrichment to saturate, and the final proportion of molecules enriched with ^13^C, were remarkably similar between isoprene and *cis*-ocimene, with both reaching around 80% enrichment after 2.5 h exposure to ^13^CO_2_. This pattern appears to be a consequence of the rapid incorporation of labelled carbon into the C5 and C10 terpene substrates dimethylallyl diphosphate (DMADP) and geranyl diphosphate (GDP), respectively. The rates of incorporation observed in emissions in *E. globulus* are comparable to the rates reported for conifer species [10], but much slower than those reported for monoterpene labelling in *Q. ilex* [10,27], which saturated at around 80% after 20–30 min. Differences in leaf architecture, such as bulk leaf density, may explain this effect. The labelling of trans-caryophyllene was slower and less complete than that of C5 isoprene and C10 *cis*-ocimene, which fits with the export of isopentenyl diphosphate (IPP) from plastids into the cytosol, where carbon from the mevalonate pathway contributes to the synthesis of sesquiterpenes [25,42]. The link between the two compartments was demonstrated previously in *E. globulus*, where deuterated deoxyxylulose was incorporated into both ocimene and caryophyllene at 83% [43].

### 3.2. Identification of ^13^C Labelling

The labelling experiments reported here unequivocally confirm the incorporation of recently fixed carbon into *cis*-ocimene and trans-caryophyllene, but the extent of this incorporation can be obscured by emissions from stored sources. This is of particular significance when analysing samples by GC-MS, and when the molecular ion is absent or present only at low concentrations. To determine whether smaller ion fragments could be reliably used to examine labelling kinetics, Loreto et al. [27] tested a statistical null model that assumed a random distribution of C atoms in the terpene molecule against the observed labelling. The conclusion of the authors that all carbons were probably randomly labelled is now known not to be true, since carbon atoms are drawn from specific substrates [23,31,44,45]. However, in the absence of a suitably abundant molecular ion, the [M-CH_3_]^+.^ ion could be used to determine incorporation kinetics, providing the number of carbons in the molecule is sufficiently high. Terpene emissions from stored sources could pose a greater problem for interpretation if they are large relative to the de novo synthesised fraction, as the incorporation of the ^13^C label would need to be determined against an intense endogenous background.

The light response observed for *cis*-ocimene in a related experiment (Winters et al., unpublished data) found that *cis*-ocimene emissions were six times greater when exposed to 500 μmol m^−2^ s^−1^ PAR (15 nmol m^−2^ min^−1^) compared to darkness (2.5 nmol m^−2^ min^−1^), where dark emissions represent those from the stored pool. Thus, the light–dark emission ratio is estimated to be around 6:1, but as the temperature was 3 °C warmer in this study (with the same light level), this ratio might be slightly smaller, as the warmer temperatures may have volatilised more stored oils; therefore, a conservative ratio of 5:1 is assumed. Thus, 20% of the ^12^C signal (at saturated enrichment of the de novo fraction) could result from stored oils. Correcting for this origin of the ^12^C signal, the calculated ^13^C incorporation increased from 77% to 79%, which is within the range of error for these estimates. Using the same spectral data, but changing the emissions from stored pools to 70% of de novo synthesis, the saturated enrichment would be corrected to 93%, representing a large change. As an alternative, Ghirardo et al. [10] used PTR-MS data, assuming the labelling seen in the monoterpenes reflects that seen in isoprene, given their proximity in the biosynthetic pathway.

The presence of *iso*-valeraldehyde was confirmed in the present study by GC-MS. This compound is a reasonable candidate for the ions observed at *m*/*z* 85 and 87, particularly given previous reports of *iso*-valeraldehyde in the oil extracts of a number of eucalypt species, including *E. globulus* [36]. However, caution is warranted as the rate of labelling seems fast relative to its putative source within the leaf. The few reports on *iso*-valeraldehyde in the literature [46,47] suggest it is emitted from pyruvate decarboxylase activity on pyruvate during the catabolism of L-leucine [47,48]. If this process is indeed the source of *iso*-valeraldehyde emitted from eucalypt leaves, it seems at odds with the rapid incorporation of ^13^C into the molecule. Conversely, 2-methyl-3-buten-2-ol and various structural isomers have been reported in the literature, mostly in pines [37,38,39,49], but also in *Quercus ilex* [27]. Retention time-matching by gas chromatography was inconclusive (early eluting broad peak due to lack of cryogenic focussing), but the major ion fragments generated from an *iso*-valeraldehyde standard using PTR-MS (Figure 4A) matched those ions seen during fumigation with ^13^CO_2_ (Figure 2). Although more ion fragments were seen in the standard, it provided a better fit than methyl butanol (Figure 4B), which has not been reported to be emitted from eucalypts.

### 3.3. The Putative Importance of De Novo Synthesis

Monoterpene synthase assays were initially of limited value due to the passage of terpene oils from the ground leaves into the protein extract. Using an incremental rise in temperature, we were able to discriminate contaminant oils from those synthesised during the incubation period, revealing that only *cis*-ocimene was produced enzymatically. The optimum temperature at 50 °C was around 10 °C higher than that found in *Q. ilex* [40], but in a similar range to the optimum isoprene synthase temperature in leaves of *E. globulus* (Winters, Schnitzler, and Zimmer, unpublished data). These results confirm that a fraction of the *cis*-ocimene emitted from *E. globulus* leaves originates indeed from a de novo source. Although no other monoterpenes were found to be synthesised during the assay, the presence of low levels of de novo-synthesised monoterpenes would have been very difficult to detect against the background of the oils carried-over through protein extraction. The absence of ^13^C labelling in any other monoterpene, either emitted or stored, suggests monoterpene synthesis may be subject to temporal regulation within *E. globulus* leaves. The seasonal regulation of monoterpene synthesis has been demonstrated previously in *Quercus ilex* [40], which does not store terpenes, and in *Pinus sylvestris* [50], which both stores and synthesises terpenes. In peppermint (*Mentha x piperita* L.), monoterpene synthases are active during leaf development, but inactive in mature leaves [51,52]. The present results suggest that once oil glands have been filled during leaf expansion, the epithelial cells become either dormant or degenerate and the oil produced until that point will be the only oil present during the leaf’s two- to four-year life span. However, the biosynthesis of non-stored terpenes persists within the mesophyll for the entire life of the leaf. A similar developmental pattern is observed in Maritime pine [41]. Preliminary evidence for this pattern also comes from an unpublished comparison of the emissions of young and old juvenile leaves of *E. globulus*, where young leaves emitted eucalyptol at a magnitude two to three orders of greater than that of *cis*-ocimene, while in the aged leaf, emissions dropped to 1:1 (Winters, unpublished data).

## 4. Materials and Methods

### 4.1. Plant Material

Seedlings of *E. globulus* Labill. subsp. *globulus* were cultivated in 20 L pots containing a sterilised mix of vermiculite and loam in a ratio of 1:2 (*v*/*v*). Plants were supplied with a low phosphorous, slow-release fertiliser (Osmocote Plus™ ICL, Sydney, Australia) and maintained under ambient light with periodic shading provided on hot days. Average diurnal temperatures ranged between 21 and 32 °C in the month before the experiments and plants were watered daily. Plants were about 18 months old and 1.5–2 m high at the time of the experiment.

### 4.2. Gas Exchange System

Gas exchange measurements were made on the distal 2–3 pairs of opposite leaves on branchlets of *E. globulus*. Healthy and visually similar leaves were placed in a 0.75 L cuvette consisting of two chambers: a lower chamber in which the leaf was housed and an upper chamber though which water from a Julabo water bath (John Morris Scientific, Sydney, Australia) was circulated to control leaf temperature. Prior to placing the leaves in the cuvette, the leaf stem was wrapped in polytetrafluoroethylene (PTFE) tape to limit abrasion of the stem. Leaves were illuminated with a 250 W metal-halide lamp and held constant at 500 μmol m^−^^2^ s^−^^1^ photosynthetically active radiation (PAR) for the duration of the experiment. Leaf temperature was held at 27.9 ± 1.3 °C for all experiments. Zero-grade air (BOC Gases, Sydney, Australia, 20.5% oxygen in nitrogen) was supplied to the cuvette at 2.3 L min^−^^1^. The air was humidified to approximately 28% relative humidity via passage through a bubbler held at 4 °C. CO_2_ (BOC Gases, Sydney, Australia) of natural isotopic abundance was added to the humidified air stream to a concentration of 400 + 9 ppm in the cuvette. The exchange of H_2_O and CO_2_ was monitored with a CIRAS II infra-red gas analyser (IRGA, PP Systems, Hertfordshire, UK) sampling air before and after the cuvette. IRGAs are less sensitive to ^13^CO_2_ (Cambridge Isotope Laboratories, Inc., Tewksbury, MA, USA) than natural abundance CO_2_, but the rates of gas exchange were used primarily to monitor plant function throughout the experiment. Leaves were positioned in the cuvette on the evening before experiments commenced, with the cuvette lid open and exposed to laboratory air. The following morning, the cuvette was sealed, and light and temperature control, and air supply commenced. The plant was allowed at least 2 h were to stabilise before the beginning of the measurements. Natural carbon abundance CO_2_ was initially supplied to the leaves for the first hour of measurements, before switching to ^13^CO_2_ for 6–8 h, then reverting to natural abundance CO_2_ for a further 1–2 h.

### 4.3. BVOC Sampling and Analysis

A high-sensitivity proton transfer reaction-mass spectrometer (PTR-MS, Ionicon Analytic GmbH, Innsbruck, Austria) was used to monitor changes in the isotopic signal of terpenes. As the quadrupole mass analyser in the PTR-MS is of unit mass accuracy and resolution, it has a limited ability to resolve and identify individual BVOC components. The airflow (80 mL min^−^^1^) from the cuvette was split between the PTR-MS and charcoal-filled adsorbent cartridges, which could trap BVOC for later identification using gas chromatography–mass spectrometry (GC-MS). Samples were also collected each morning and evening from the upstream side of the cuvette in order to check for potential background interferences (always negligible). Protonated parent and fragment ions of isoprene (*m*/*z* 69 to 74) and monoterpenes (*m*/*z* 81 to 95; 135 to 145; 155 to 165) were monitored using PTR-MS in selected ion mode (SIM), with the drift tube operated at 126 Td and held at 40 °C. The instrument was calibrated daily with an eight-component standard gas mix (Apel Reimer Environmental, Denver, CO, USA) and transmission was determined weekly using liquid standards diluted in Tedlar bags.

Custom-made adsorbent charcoal cartridges collected gas-phase monoterpenes for the subsequent analysis of isotopic incorporation using GC-MS. Borosilicate glass tubes (7 cm × 3.2 mm id) were packed with 5 mg of 20–60 mesh activated charcoal (Sigma Aldrich, Sydney, Australia) and plugged at either end with silanised glass wool. The charcoal was pre-treated by heating to 450 °C while passing helium through the bed at 80 mL min^−^^1^ for 4 h. During sampling, air was drawn through the cartridge at 0.08 L min^−^^1^ for 60 min. Trapped terpenes were solvent-extracted by transferring the charcoal bed to a 2 mL glass vial containing a 300 μL insert. Carbon disulphide (50 μL) was added and allowed to stand for 30 min at room temperature when the vial was centrifuged and the supernatant was transferred to a second vial for storage at 4 °C before analysis by GC-MS. A pilot study indicated that extraction times greater than 30 min did not improve analyte recovery and analytes were found to be stable for up to eight weeks.

### 4.4. Extraction of Terpenes from Individual Oil Glands and Leaf Tissue

PTR-MS revealed that the incorporation of ^13^C into emitted terpenes reached a maximum after ca. 6 h. To determine if ^13^C was taken up by terpenes remaining within the leaf tissue, a further four studies were carried out where leaves were removed from the cuvette immediately, after 6–8 h of fumigation with ^13^CO_2_. Of the four leaves, two were stored in liquid N_2_ for the later extraction of terpene oils from homogenised leaf tissue. Leaves were ground under liquid N_2_, and 100 mg (fresh weight) was placed in a vial containing 2 mL of chilled pentane spiked with 100 μg mL^−^^1^ 1,2,4-trimethylbenzene as an internal standard. The extracts were allowed to stand at room temperature for four days before the liquid was drawn off for subsequent analysis by GC-MS.

Individual oil glands were sampled on the remaining two leaves using a glass capillary previously drawn to a fine point with a custom-made capillary puller. A bottom-lit dissecting microscope was used to locate the subdermal oil glands and position the capillary tip. A Petri dish containing chilled water was used as a stage to allow the light to pass up into the leaf while limiting the transfer of heat to the leaf. A syringe connected via flexible tubing to the capillary was used to draw oil from the pierced gland into the tip of the capillary. Once 30–40 glands across the leaf had been sampled, the tip was rinsed in a 2 mL vial containing a 300 µL insert filled with 40 µL of chilled pentane. A pilot study demonstrated no variation in oil composition between glands within a given leaf.

### 4.5. GC-MS Analysis of Individual Terpenes

Aliquots of 1 µL were injected via an autosampler into a Polaris Q GC-MS (Thermo Fisher Scientific, Sydney, Australia) (injection port 220 °C; interface 230 °C; source 200 °C). Chromatographic separation was achieved using a SolGel Wax fused–silica capillary column (SGE Analytical Science, Melbourne, Australia; 30m × 0.25 mm id, 0.25 µm phase thickness) with He as the carrier gas (inlet pressure 96.5 kPa). The temperature programme began at 30 °C and was held for 0.5 min; increased to 150 °C at 5 °C min^−^^1^; and increased to 240 °C at 15 °C min^−^^1^ and was held for 2 min. The mass spectrometer was operated in the electron ionisation (EI) mode with an ionisation energy of 70 eV. Mass spectra were acquired with full scans from 50 to 250 u in 0.33 s. Peaks were identified through comparing retention time and fragmentation spectra (MS similarity > 80% with authentic standards), and where these were not available, through comparison with the NIST-Wiley Mass Spectra Library (1997) (MS similarity > 80%).

### 4.6. Monoterpene Synthase Assay

Leaves from the trees used in the gas exchange study, in addition to similarly aged leaves from specimens of *E. viminalis,* grown under identical conditions in Australia, were assayed for monoterpene synthase (mono-TPS) activity according to the protocol described in [40,53]. Healthy juvenile leaves were instantly frozen in liquid N_2_ and packed in solid CO_2_ for transport. Upon arrival, they were stored at −80 °C until being assayed, approximately four weeks later. Proteins were isolated using the modified protocols previously developed for *Quercus ilex* [40]. The terpene synthase activity protocols developed for *Q. ilex* suffered from interference from the terpene oils, which had been carried into the protein extract, and various attempts to separate the oil from the extract proved unsuccessful. The enzyme activity was therefore assayed using temperature response to identify mono-TP synthase activity above the background level.

For the temperature response assay, leaves were ground under liquid N_2_ and 100 mg (fresh weight) of the powder was suspended in 5 mL of chilled extraction buffer (0.7 M MOPS, pH 7.3, 1.5% PEG, 20 mM MgCl_2_, 1% PVP-30 (*w*/*v*), 8.3% (*w*/*v*) Dowex 1 × 2, 0.5 M Na-ascorbate, 0.5 M Na_2_S_2_O_5_, 0.5 M DTT). The remaining steps in the protocol were conducted at 4 °C. After stirring for 15 min, the homogenate was centrifuged at 18,000× *g* for 20 min and 2.5 mL of the supernatant was desalted on PD-10 columns (Amersham-Pharmacia, Freiburg, Germany) with elution buffer (50 mM KPi, pH 7.3, 10% glycerol (*v*/*v*) and antioxidants (200 mM Na-ascorbate and 50 mM β–mercaptoethanol). From the resulting 3.5 mL of protein extract, 93 μL was transferred into 2 mL gas-tight crimp vials, followed by 2 μL of 1 M MgCl_2_. The enzyme reaction was started by adding 5 μL of 5 mM geranyl diphosphate (GDP) (final concentration: 250 μM) and vials were incubated for 60 min at one of eight 5 °C temperature increments between 25 °C and 60 °C. The reaction was stopped by removing the reaction mixture with a syringe and rinsing the vial with 150 μL of distilled water. Monoterpenes generated through the reaction of mono-TPS with the GDP substrate were sampled from the headspace and analysed via gas chromatography (GC).

A CP-9000 gas chromatograph (Chrompack, Frankfurt/M., Germany) equipped with a headspace-volume-autosampler (Gerstel, Mülheim, Germany) and temperature-programmed injection system (KAS 3, Gerstel, Mülheim, Germany) was used for analysis (40). Headspace injections of 1 mL were concentrated on a precolumn trap containing Tenax TA (60/80 mesh) at 27 °C. Trapped compounds were desorped at 240 °C onto a capillary column (DB-1701, 30 m × 0.25 mm I.D., 1 μm film thickness; J&W Scientific, Folsom, CA, USA). The column was then temperature-programmed from 35 °C (held for 0.5 min) to 78 °C at 30 °C min^−^^1^ (held for 4 min), then increased to 160 °C at 9 °C min^−^^1^, and then to 250 °C at 35 °C min^−^^1^. Products were detected with an FID operating at 270 °C. Compounds were identified through a retention time comparison with authentic standards. Protein extracts incubated without the GDP substrate were used for background correction of the terpene signals.

### 4.7. Data Analysis and Error Determination

Error estimates incorporating calibration and sampling errors were calculated through the propagation of errors [54]. The half-life time of the decay of the unlabelled signal of compounds detected by PTR-MS was determined from the time evolution of this signal according to the equations N(t) = N_0_ e^−λτ^ and τ(1/2) = ln(2)/λ, where N(t) is the emission rate at time t, N_0_ is the calibrated instrument signal at t = 0, and λ is an empirically determined decay constant. Constants within each function were determined by fitting the function to each set of normalised data points and constraining it until an acceptable fit was achieved (*R*^2^ > 90%).” Half-life time calculations were made using GraphPad Prism 5 (GraphPad Software, San Diego, CA, USA).

## 5. Conclusions

Apart from isoprene, previous studies of eucalypts suggest that oil glands dominate terpene emissions that are mainly regulated by temperature. Given the general predominance of oil glands in eucalypts, these are logical conclusions. Here, we show that while stored pools account for most terpenes emitted from juvenile leaves of *E. globulus*, the monoterpene *cis*-ocimene and the sesquiterpene trans-caryophyllene originate from de novo synthesis. In addition to isoprene, an oxygenated C5 compound identified as iso-valeraldehyde was also emitted directly from de novo synthesis. Future studies need to identify how these emissions respond to light and temperature, given the preponderance of high-light, high-temperature eucalypt habitats. Similarly, further attention is required for the identification and quantification of iso-valeraldehyde, as well as to determine its response to environmental variables. This need is underscored by the large fragments produced by iso-valeraldehyde at *m*/*z* 69, which may result in serious errors in the estimation of isoprene fluxes.

## Figures and Tables

**Table 1:** The relative chromatographic abundance of 34 terpene compounds in the oil glands and whole leaf of juvenile *E. globulus* under light and dark conditions (*n* = 4). Numbers in front of the compound name indicate compounds positively identified against authentic standards. Compounds **7** and **11** undergo de novo synthesis during emission. See Figure 1 for GC-MS chromatogram. Unnumbered compounds identified by chemical formula had mass spectra indicative of terpenes. * Note that 1,8-cineole is also known as eucalyptol.

		Oil Glands				Whole Leaf			
Compound	RT	Light		Dark		Light		Dark	
	(min)	Mean	SE	Mean	SE	Mean	SE	Mean	SE
1. α-pinene	4.16	8.43	±0.45	9.62	±0.59	9.44	±0.34	8.82	±0.36
2. β-pinene	5.78	0.49	±0.02	0.52	±0.01	0.59	±0.02	0.52	±0.02
3. R-(-)-α-phellandrene	7.17	0.06	±0.01	0.06	±0.01	0.21	±0.15	0.07	±0.01
4. myrcene	7.31	0.93	±0.06	0.97	±0.04	1.13	±0.08	1.01	±0.04
5. limonene	8.04	2.96	±0.09	3.09	±0.09	2.72	±0.09	3.12	±0.20
6. 1,8-cineole *	8.22	59.1	±0.90	58.55	±1.21	56.43	±0.79	58.31	±3.18
7. *cis*-ocimene	9.10	0.23	±0.04	0.14	±0.04	0.28	±0.05	0.32	±0.02
8. *p*-cymene	9.26	0.32	±0.03	0.34	±0.03	0.31	±0.02	0.35	±0.03
9. *trans*-ocimene	10.2	0.04	±0.01	0.04	±0.01	0.05	±0.01	0.04	±0.01
C_10_H_16_	10.74	0.07	±0.01	0.08	±0.02	0.07	±0.01	0.06	±0.01
10. *p*-α-dimethylstyrene	14.5	0.02	±0.01	0.02	±0.01	0.03	±0.01	0.02	±0.01
C_15_H_24_	14.93	0.03	±0.01	0.03	±0.01	0.05	±0.01	0.04	±0.01
C_15_H_24_	15.134	0.60	±0.03	0.57	±0.04	0.60	±0.03	0.59	±0.06
C_15_H_24_	15.53	0.37	±0.06	0.35	±0.07	0.56	±0.07	0.45	±0.11
C_15_H_24_	15.85	0.02	±0.01	0.03	±0.01	0.03	±0.01	0.03	±0.01
C_15_H_24_	16.36	0.01	±0.01	0.01	±0.01	0.01	±0.01	0.01	±0.01
C_10_H_16_O	16.72	4.43	±0.25	4.19	±0.36	4.70	±0.22	4.55	±0.45
C_15_H_24_	16.97	0.23	±0.01	0.22	±0.02	0.26	±0.01	0.24	±0.03
C_15_H_24_	17.21	0.04	±0.01	0.05	±0.01	0.08	±0.01	0.05	±0.01
C_15_H_24_	17.40	0.05	±0.01	0.04	±0.01	0.05	±0.01	0.04	±0.01
C_15_H_24_	17.54	0.02	±0.01	0.02	±0.01	0.04	±0.01	0.03	±0.01
C_15_H_24_	17.65	0.06	±0.01	0.06	±0.01	0.07	±0.01	0.06	±0.01
C_15_H_24_	17.88	0.01	±0.01	0.01	±0.01	0.02	±0.01	0.02	±0.01
11. *trans*-caryophyllene	18.24	0.65	±0.03	0.63	±0.05	0.73	±0.03	0.71	±0.01
C_15_H_24_	18.43	0.12	±0.01	0.12	±0.01	0.13	±0.01	0.13	±0.01
12. aromadendrene	18.612	5.37	±0.28	5.10	±0.28	4.90	±0.38	5.47	±0.02
C_15_H_24_	18.72	0.12	±0.01	0.11	±0.01	0.10	±0.01	0.12	±0.63
C_15_H_24_	18.83	0.24	±0.01	0.22	±0.02	0.24	±0.02	0.24	±0.02
C15H24	19.23	0.29	±0.01	0.30	±0.02	0.31	±0.01	0.30	±0.03
13. alloaromadendrene	19.54	1.61	±0.36	1.78	±0.33	1.78	±0.23	2.42	±0.04
C_15_H_24_	19.8	0.69	±0.36	0.72	±0.39	0.75	±0.40	0.54	±0.05
C_15_H_24_	19.99	0.19	±0.02	0.15	±0.02	0.17	±0.03	0.22	±0.30
C_15_H_26_O	20.11	0.06	±0.03	0.05	±0.02	0.06	±0.02	0.05	±0.01
14. a-terpineol	20.02	0.01	±0.01	0.02	±0.01	0.02	±0.01	0.02	±0.02

## Data Availability

Raw data are available from the authors on request.

## Abbreviations

The following abbreviations are used in this manuscript:

BVOC	Biogenic volatile organic compounds
DMADP	Dimethylally diphosphate
GC-MS	Gas chromatography–mass spectrometry
GDP	Geranyl diphosphate
MVA	Mevalonic acid
MEP	Methylerithritol phosphate
PAR	Photosynthetically active radiation
PTR-MS	Proton transfer reaction–mass spectrometer
RT	Retention time
SIM	Selected ion monitoring
TPS	Terpene synthase
